# Haemophagocytic syndrome and elevated EBV load as initial manifestation of Hodgkin lymphoma in a HIV patient: case report and review of the literature

**DOI:** 10.7448/IAS.17.4.19650

**Published:** 2014-11-02

**Authors:** Delphine Sculier, Thanh Doco-Lecompte, Mathieu Rougemont, Alexandra Calmy

**Affiliations:** Infectious Diseases, University Hospitals of Geneva, Geneva, Switzerland

## Abstract

**Introduction:**

In HIV patients, haemophagocytic syndrome (HPS) may occur in the presence of cancer, concomitant viral infection, HIV primo-infection or at the initiation of highly active antiretroviral therapy (HAART). Hodgkin lymphoma remains a rare cause of HPS. We describe a case of HPS with very high Epstein Barr virus (EBV) load in a HIV patient as initial manifestation of Hodgkin lymphoma.

**Materials and Methods:**

A 29-year-old HIV positive man, successfully treated with HAART with an undetectable viral load and CD4 cells count of 438/µl, was admitted for high fever of unknown origin. Laboratory results showed a pancytopenia with haemoglobin at 82 g/l, lymphocyte count at 0.36G/l and platelets count at 47G/l; a highly elevated ferritine >7500 µg/l; increased lactate dehydrogenase at 885U/l and soluble IL2 receptor (CD25) >60 ng/ml. EBV load was measured and confirmed at 2,600,000 copies/ml. A PET-CT imaging showed diffuse elevated metabolic activity in the bone marrow and in two lesions in the spleen without lymphadenopathy. Bone marrow and liver biopsies revealed images of haemophagocytosis and lymphocyte depleted Hodgkin lymphoma. Treatment consisted in etoposid, steroids, and R-ABVD (rituximab, doxorubicin, bleomycin, vinblastine, dacarbazine) chemotherapy. The patient completed six cycles of chemotherapy. We reviewed the literature in PubMed with the following keywords: HPS, HIV, EBV, Hodgkin lymphoma.

**Results:**

We identified four publications and two reviews reporting cases of HPS associated with Hodgkin lymphoma in HIV patients with either a positive EBV load either the presence of encoded EBV RNA in tumour cells. Twenty-two cases (including one pediatric case) were described. Among adults, mostly men, the median age was <50 years and immune suppression was marked with a median CD4 cell count<100 cells/µl, even in patients receiving HAART. When measured, EBV load in the serum was high. Prognosis was poor with a high mortality despite adequate treatment consisting in steroids and chemotherapy, with or without etoposide (Table 1).

**Conclusions:**

Our case report and the review of literature suggest that physicians should be aware of the association between EBV infection/reactivation and Hodgkin lymphoma as a cause of HPS in HIV patients, even if successfully treated with HAART. The pathogenesis of these three interrelated conditions (viral infection, oncogenesis and immunologic reaction) remains unclear.

**Table 1:** Reported cases of HPS associated with Hodgkin lymphoma and high EBV load in HIV patients

Case report	Number of cases	Sex	Age (years)	CD4 count/µl	Encoded EBV RNA in tumour cells	PCR EBV in serum/ml	Evolution
Khagi et al. (Clin Adv Hematol Oncol 2012)	1	M	58	314	n/a	54,954	Died
Flew et al. (Int J STD AIDS 2010)	1	M	46	40	Positive	27,000	Alive
Preciado et al. (Leuk Lymphoma 2001)	1	M	8	90	Positive	n/a	Died
Albrecht et al. (Arch Pathol Lab Med 1997)	1	M	26	n/a	Positive	n/a	Died
Review	Number of cases	Ratio M/F	Median age (years)	Median CD4 count/µl	Encoded EBV RNA in tumor cells	PCR EBV in serum/ml	Evolution
Fardet et al. (AIDS 2010) α	10	n/a	42 α	91	n/a	20,000 α	Not favourable α
Ménard et al. (Clin Infect Dis) β	8	3:1 β	38 β	n/a	100% β	n/a	n/a

Legend: n/a: not available; α Review of 58 HPS cases, 10 associated with Hodgkin lymphoma, reported values related to the 58 cases; β Review of 34 HPS cases associated with Hodgkin lymphoma, 8 in HIV patients, reported values related to the 8 HIV cases.

